# Large-Scale Synchronization Dynamics During Epileptic Seizures: A Patient-Independent EEG Network Analysis

**DOI:** 10.3390/e28060599

**Published:** 2026-05-27

**Authors:** Oleg Gorshkov, Hernando Ombao

**Affiliations:** Statistics Program, King Abdullah University of Science and Technology, Thuwal 23955, Saudi Arabia; hernando.ombao@kaust.edu.sa

**Keywords:** epileptic seizures, EEG functional connectivity, phase synchronization, patient-independent classification, leave-one-patient-out (LOPO) validation

## Abstract

This study examines large-scale synchronization dynamics during epileptic seizures using scalp EEG recordings, with the aim of characterizing reproducible network-level patterns across patients. Functional connectivity was estimated from the CHB-MIT database using phase-lag-based measures robust to volume conduction, specifically Imaginary Coherence and the debiased weighted phase lag index, across standard frequency bands. Synchronization features were used to train a neural network classifier evaluated under a Leave-One-Patient-Out (LOPO) validation framework to ensure patient-independent assessment. To quantify seizure-related network alterations, we introduce Relative Pathological Synchronization (RPS), defined as the median area under the ROC curve across patients. The results demonstrate that synchronization patterns deviate systematically from baseline activity in a time-dependent manner. Interhemispheric connectivity shows earlier and higher peak RPS values compared to intrahemispheric connectivity, while intrahemispheric changes develop more gradually and persist over a longer interval. Theta-band features provide the most consistent contribution, although interhemispheric synchronization involves multiple frequency bands. In addition, longer seizures are associated with higher peak RPS values. These findings indicate that large-scale synchronization patterns contain stable, patient-independent information about seizure dynamics. Specifically, interhemispheric connectivity achieved a peak RPS of 0.749 (0.609–0.891) at TAS=10 s, while intrahemispheric connectivity reached 0.640 (0.563–0.843) at TAS=30 s under strict Leave-One-Patient-Out validation.

## 1. Introduction

Epilepsy is one of the most common chronic neurological disorders, affecting more than 50 million people worldwide and characterized by recurrent, unprovoked seizures resulting from abnormal neuronal activity in the brain [1,2]. Traditionally, epilepsy was conceptualized as a disorder caused by localized hyperexcitable regions known as epileptic foci. However, accumulating evidence from electrophysiology, neuroimaging, and computational neuroscience has led to a paradigm shift toward understanding epilepsy as a disorder of large-scale brain networks rather than a purely focal pathology [3,4]. Within this framework, seizures emerge from abnormal interactions among distributed neuronal populations that dynamically evolve over time.

A central feature of epileptic seizures is abnormal neuronal synchronization. Early experimental and clinical studies demonstrated that seizures often involve excessive coordination of neuronal firing across cortical and subcortical regions, producing the hypersynchronous electrical activity observed in electroencephalography (EEG) recordings [1,2]. At the same time, recent work suggests that seizure dynamics cannot be explained solely by simple hypersynchrony; rather, seizures involve complex spatiotemporal patterns of synchronization and desynchronization across interacting neural networks [2]. This evolving perspective has motivated extensive investigation of functional connectivity in epileptic brain networks.

Functional connectivity analysis aims to quantify statistical dependencies between signals recorded from different brain regions. EEG-based connectivity measures have been widely used to study the interactions underlying seizure initiation, propagation, and termination [1,5]. Such approaches have revealed that seizure evolution is accompanied by systematic changes in network topology, including alterations in clustering, path length, and modular organization of functional brain networks [6,7]. In particular, several studies have reported increased synchronization between cortical areas during ictal activity, suggesting that large-scale coordination among neuronal populations plays a crucial role in seizure generation and maintenance [8,9].

Connectivity analysis in EEG data presents important methodological challenges. One major issue is volume conduction, whereby electrical activity from a single neural source can propagate through brain tissue and be simultaneously recorded by multiple electrodes, artificially inflating measures of connectivity based on instantaneous correlations [10,11]. To address this limitation, phase-lag-based connectivity metrics have been developed that selectively capture delayed interactions between brain regions. Measures such as Imaginary Coherence and the Phase Lag Index suppress zero-phase coupling and therefore provide more reliable estimates of true physiological interactions between neuronal populations [10,11,12].

In parallel with advances in connectivity analysis, machine learning approaches have increasingly been applied to EEG data for seizure detection, prediction, and characterization. Algorithms ranging from classical classifiers to deep neural networks have demonstrated promising performance in identifying seizure states and localizing epileptogenic regions [13,14]. More recently, network-based machine learning models have been proposed to integrate connectivity features derived from multi-channel EEG recordings, allowing the detection of complex patterns of large-scale brain interactions [7].

A substantial body of work has applied machine learning to EEG-based seizure detection on the CHB-MIT database, ranging from classical feature engineering with shallow classifiers to end-to-end deep neural network architectures. Liu et al. [15] proposed a channel-perturbation convolutional neural network combined with a bidirectional long short-term memory (BiLSTM) network and evaluated it in a strictly patient-independent (cross-patient) setting, reporting mean sensitivity 86.51% and average AUC-ROC of 90.82%, respectively. Tian et al. [16] combined brain connectivity features derived from the phase-locking value and Pearson correlation with a CNN-Transformer classifier, achieving detection accuracy above 99% on CHB-MIT; however, their evaluation followed a patient-specific protocol. Chen et al. [17] introduced an explainable statistical framework based on functional connectivity features, obtaining an AUC of 0.940 and sensitivity of 93.0% under a patient-specific validation scheme. Carzaniga et al. [18] proposed CA-EEGWaveNet, a channel-adaptive architecture evaluated under a LOPO scheme, surpassing the baseline EEGWaveNet with a median F1-score of 0.78.

Despite these advances, the majority of high-performing systems optimise segment-level detection metrics under either patient-specific training or mixed-patient batches, and relatively few studies are evaluated under a strict Leave-One-Patient-Out (LOPO) scheme that entirely excludes one patient from both model training and feature normalisation. Furthermore, existing methods rarely characterise the temporal evolution. of synchronisation patterns relative to seizure onset, nor do they provide a compact, population-level indicator of pathological network organisation.

The present study addresses a complementary objective: rather than maximising binary detection performance for clinical deployment, we aim to characterise *how* and *when* large-scale synchronisation patterns deviate from baseline activity across patients. To this end we introduce Relative Pathological Synchronisation (RPS), a model-derived indicator of population-level separability evaluated under a strict LOPO validation framework. A comparison of the proposed approach with contemporary patient-independent methods is provided in Discussion.

A key unresolved question concerns how large-scale neuronal synchronization evolves during seizures and whether these patterns exhibit consistent organization across individuals. In particular, the relative contributions of intrahemispheric and interhemispheric interactions remain incompletely understood. In the present study, we investigate large-scale synchronization dynamics during epileptic seizures using EEG recordings from the CHB-MIT database [19,20]. Functional connectivity was quantified using phase-lag-based measures robust to volume conduction, namely Imaginary Coherence and the Debiased Weighted Phase Lag Index. Instead of interpreting connectivity values directly, we introduce a model-derived indicator termed *Relative Pathological Synchronization* (RPS), defined as the Area Under the ROC Curve obtained from neural network models trained to discriminate seizure-related intervals from baseline activity under a Leave-One-Patient-Out (LOPO) validation framework.

The main objective of this work is to characterize how synchronization patterns evolve during seizures and the extent of reproducibility of these patterns across patients. Specifically, we aim to:

- Quantify seizure-related synchronization using phase-lag-based connectivity metrics;
- Compare intrahemispheric and interhemispheric synchronization patterns;
- Evaluate inter-patient generalizability using a LOPO validation scheme;
- Investigate the relationship between synchronization reproducibility and seizure duration.

By integrating functional connectivity analysis with machine learning in a patient-independent evaluation framework, this study provides a systematic characterization of large-scale synchronization dynamics underlying epileptic seizures. The remainder of this paper is organized as follows. Section 2 describes the materials and methods, including the dataset, preprocessing pipeline, connectivity metrics, feature extraction, labelling strategy, validation scheme, and the definition of RPS. Section 3 presents the experimental results, covering the temporal evolution of RPS, the relationship between seizure duration and peak RPS, feature importance, and statistical comparisons across frequency bands. Section 4 discusses the findings in the context of existing literature, and Section 5 concludes the paper.

## 2. Materials and Methods

This section describes the analysis pipeline used to characterise large-scale synchronisation dynamics during epileptic seizures. The pipeline consists of six main stages, outlined here for clarity before each is described in detail. (i) EEG recordings from the CHB-MIT database are preprocessed and segmented into non-overlapping 5-second windows; a fixed subset of 12 scalp channels is retained to ensure spatial consistency across patients (Section 2.1 and Section 2.2). (ii) Two phase-lag-based connectivity metrics—Imaginary Coherence (ImagCoh) and the Debiased Weighted Phase Lag Index (dwPLI)—are computed for six interhemispheric and six intrahemispheric channel pairs across four canonical frequency bands (δ, θ, α, β), yielding a 24-dimensional feature vector per window (Section 2.3 and Section 2.4). (iii) Each window is labelled according to its temporal offset relative to seizure onset, parametrised by the Time After Seizure onset (TAS); baseline activity is defined from a temporally distant pre-onset interval $B=[t_{\mathrm{onset}}-600,\;t_{\mathrm{onset}}-30]$ s (Section 2.5). (iv) A shallow multilayer perceptron (MLP; architecture 24–64–2) is trained to discriminate seizure-related windows from baseline, with features standardised via z-score normalisation using training-set statistics only (Section 2.6). Model generalisation is assessed under a strict Leave-One-Patient-Out (LOPO) cross-validation scheme, in which each patient is held out in turn from both training and feature normalisation (Section 2.7). (vi) Classification performance is summarised by the Area Under the ROC Curve (AUC-ROC); its mean across LOPO folds defines the Relative Pathological Synchronisation (RPS(TAS))—a compact, patient-independent indicator of how consistently seizure-related network patterns deviate from baseline as a function of time relative to onset (Section 2.8).

### 2.1. Dataset

The dataset used in this study is the CHB-MIT scalp EEG database available on PhysioNet [19,20]. This EEG Database contains electroencephalographic (EEG) recordings of pediatric patients with intractable epilepsy. The data were collected at the Children’s Hospital Boston during long-term monitoring following withdrawal of anti-seizure medication in order to capture epileptic seizures and evaluate the patients for possible surgical treatment. The dataset includes recordings from 22 subjects grouped into 23 cases. Each case consists of multiple EDF files containing continuous EEG segments, typically about one hour long. The signals were digitized at a sampling frequency of 256 Hz with 16-bit resolution. Most recordings contain 23 EEG channels placed according to the international 10–20 electrode placement system. In total, the database contains 664 EEG recordings, of which 129 files include epileptic seizures, with 198 seizures annotated in total. For these recordings, annotation files provide the start and end times of each seizure relative to the beginning of the recording.

In this study, only EEG recordings containing seizures were used for synchronization analysis. The provided seizure annotations were used to identify and extract the relevant signal segments.

### 2.2. EEG Preprocessing

Continuous scalp EEG recordings were analyzed using a window-based preprocessing pipeline designed for subsequent synchronization analysis. All EEG recordings were sampled at $f_{s}=256\;\mathrm{Hz}$. The signals were segmented into non-overlapping windows of duration 5s, corresponding to $N_{\mathrm{samp}}=1280$ samples per window. A fixed subset of 12 EEG channels was selected in order to ensure spatial consistency across recordings and patients. The selected channels were:

Fp1,Fp2,F7,F8,T3,T4,T5,T6,O1,O2,F3,F4.

**Figure 1:**
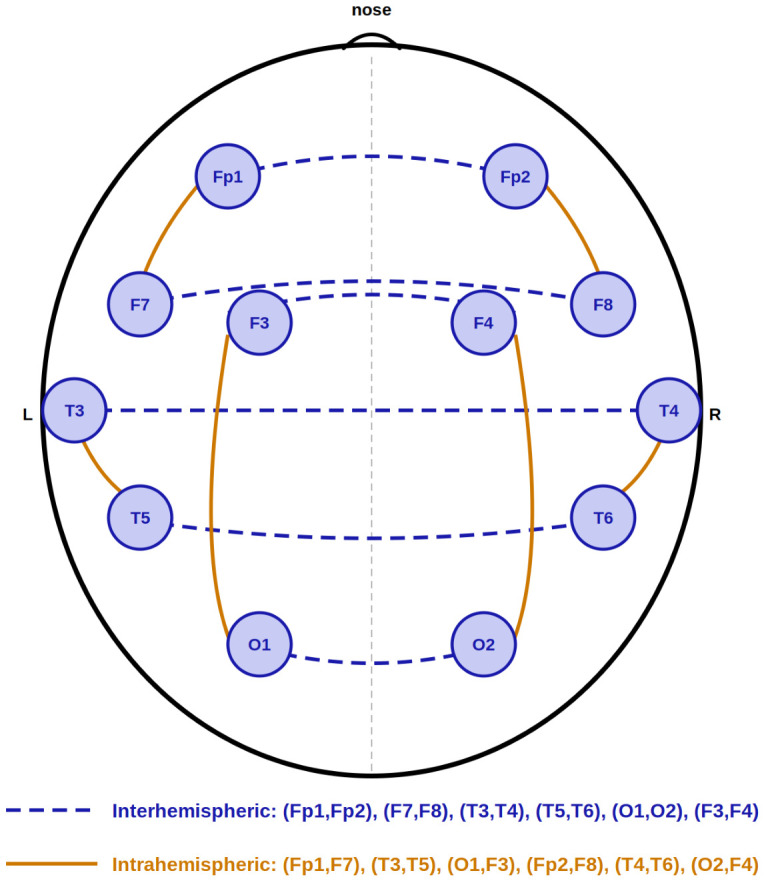
The location of the electrodes.

Figure 1 illustrates the location of the corresponding electrodes.

The use of a reduced channel subset was motivated by the fact that some recordings within the dataset did not contain a larger number of consistently available or artifact-free channels. Restricting the analysis to a common subset of channels allowed inclusion of a larger number of seizure recordings across patients. Prior to spectral analysis, each EEG window was multiplied by a Hanning taper:

$$x_{w}\left(t\right)=x\left(t\right)\;w\left(t\right),$$

where w(t) denotes the Hanning window function. Window tapering was applied to reduce spectral leakage during subsequent frequency-domain analysis. After tapering, spectral representations were computed using the discrete Fourier transform (DFT) implemented via a fast Fourier transform (FFT) algorithm for real-valued signals. The resulting spectral coefficients were subsequently used for computation of phase-based connectivity measures described in the following section.

### 2.3. Connectivity Metrics

To characterize functional interactions between spatially distributed EEG channels, phase-based synchronization measures were computed in the frequency domain. Synchronization analysis was performed separately within four canonical EEG frequency bands:

δ(0.5–4Hz),θ(4–8Hz),α(8–13Hz),β(13–30Hz).

Connectivity was estimated between predefined channel pairs representing interhemispheric and intrahemispheric interactions. Interhemispheric synchronization was computed between six homotopic channel pairs:

(Fp1,Fp2),(F7,F8),(T3,T4),(T5,T6),(O1,O2),(F3,F4).

These pairs correspond to anatomically homologous cortical regions across the two hemispheres and were selected to characterize symmetric interhemispheric functional connectivity. The montage samples frontal, temporal, and occipital cortical areas, enabling spatially distributed assessment of bilateral synchronization dynamics. Homotopic interhemispheric connections are known to reflect transcallosal communication and are therefore particularly relevant for investigating large-scale synchronization and seizure propagation mechanisms. Intrahemispheric synchronization was computed between six ipsilateral channel pairs:

(Fp1,F7),(T3,T5),(O1,F3),(Fp2,F8),(T4,T6),(O2,F4).

These pairs were selected to represent functionally distinct cortical regions within each hemisphere, including frontal, temporal, and occipito-frontal pathways. The montage incorporates both short- and long-range intrahemispheric connections and preserves left–right symmetry, enabling comparative assessment of hemispheric synchronization dynamics while minimizing the influence of interhemispheric coupling.

For each EEG window, signals were multiplied by a Hanning taper prior to spectral analysis in order to reduce spectral leakage. The discrete Fourier transform was then computed using a fast Fourier transform (FFT) algorithm. Let x(t) and y(t) denote two EEG signals within a given analysis window. Their Fourier transforms are defined as

(1) X(f)=FFT(x(t)w(t)),Y(f)=FFT(y(t)w(t)),

where w(t) denotes the Hanning taper. The cross-spectrum between the two signals is defined as

(2) $$S_{XY}\left(f\right)=E\left[X\left(f\right)Y^{*}\left(f\right)\right],$$

where ^*^ denotes complex conjugation and E[·] denotes spectral averaging. Similarly, the auto-spectra are defined as

(3) $$S_{XX}\left(f\right)=E\left[{\left|X\left(f\right)\right|}^{2}\right],\;S_{YY}\left(f\right)=E\left[{\left|Y\left(f\right)\right|}^{2}\right].$$

In practice, spectral quantities were averaged across frequency bins within each frequency band Ω. Two phase-based connectivity metrics were computed for each channel pair and frequency band: Imaginary Coherence (ImagCoh) and Debiased Weighted Phase Lag Index (dwPLI).

#### 2.3.1. Imaginary Coherence

Imaginary Coherence was used to estimate non-zero phase-lag synchronization while reducing the influence of volume conduction and common reference effects [10].

For a frequency band Ω, Imaginary Coherence was computed as

(4) $$\mathrm{ImagCoh}\left(\Omega \right)=\frac{\left|\mathrm{Imag}\left(\frac{1}{K}\sum_{f\in \Omega }S_{XY}\left(f\right)\right)\right|}{\sqrt{\left(\frac{1}{K}\sum_{f\in \Omega }S_{XX}\left(f\right)\right)\left(\frac{1}{K}\sum_{f\in \Omega }S_{YY}\left(f\right)\right)+\varepsilon }},$$

where Imag(·) denotes the imaginary part, *K* is the number of frequency bins within the band, and ε is a small positive constant introduced for numerical stability.

Imaginary Coherence (ImCoh) quantifies the strength of non-zero phase-lag synchronization between two signals in the frequency domain [10,21]. Since only the imaginary component of the cross-spectrum is considered, ImCoh effectively suppresses instantaneous correlations arising from volume conduction and common reference effects. Consequently, elevated ImCoh values are generally interpreted as evidence of physiologically meaningful delayed interactions between spatially separated cortical regions.

**Figure 2:**
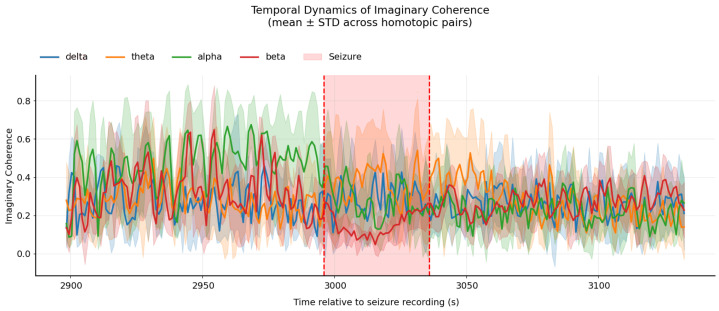
Temporal dynamics of ImCoh for four frequency bands during an epileptic seizure.

Figure 2 shows temporal dynamics of Imaginary Coherence (mean ± STD across interhemispheric homotopic pairs) for four frequency bands during an epileptic seizure (red shaded area indicates the clinically annotated ictal period). The analyzed window covers 100 s before seizure onset, the entire seizure, and 100 s after termination.

Figure 2 demonstrates a pronounced decrease in Imaginary Coherence within the beta frequency band during the ictal period. Beta oscillations are commonly associated with long-range cortical communication, cognitive integration, and organized interregional interactions [3,22]. Therefore, the observed reduction in beta-band ImCoh suggests a disruption of physiologically coordinated fast interhemispheric communication during seizure activity. Such a decrease may reflect the breakdown of normal large-scale functional integration caused by hypersynchronous epileptic dynamics.

#### 2.3.2. Debiased Weighted Phase Lag Index

To further quantify phase-lag consistency while reducing the influence of noise and sample-size bias, the debiased weighted phase lag index (dwPLI) was computed [23]. For each frequency *f*, define

$$I\left(f\right)=\mathrm{Imag}\left(S_{XY}\left(f\right)\right)$$

The dwPLI for a frequency band Ω is defined as

(5) $$\mathrm{dwPLI}\left(\Omega \right)=\frac{{\left(\sum_{f\in \Omega }I\left(f\right)\right)}^{2}-\sum_{f\in \Omega }I{\left(f\right)}^{2}}{{\left(\sum_{f\in \Omega }\left|I\left(f\right)\right|\right)}^{2}-\sum_{f\in \Omega }I{\left(f\right)}^{2}}.$$

This formulation follows the bias correction proposed by Vinck et al. [23], where frequency bins within the selected band are treated analogously to independent observations. All spectral quantities are computed separately for each frequency band Ω∈{δ,θ,α,β}.

The debiased Weighted Phase Lag Index (dWPLI) measures the consistency and temporal stability of non-zero phase differences between signals [23]. High dWPLI values indicate that one signal systematically leads or lags another while maintaining a stable phase relationship over time. As a result, dWPLI is particularly sensitive to persistent phase synchronization and is comparatively robust against noise and volume conduction artifacts.

**Figure 3:**
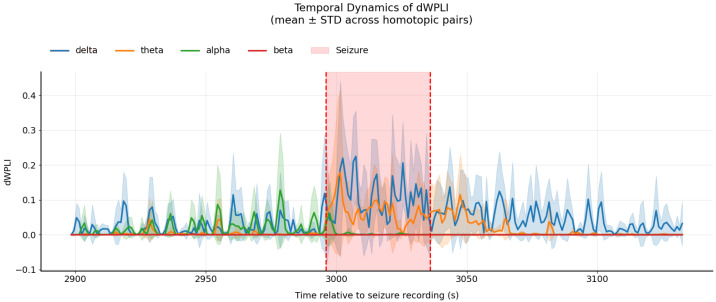
Temporal dynamics of dWPLI for four frequency bands during an epileptic seizure.

Figure 3 demonstrates temporal dynamics of dWPLI (mean ± STD across interhemispheric homotopic pairs) for four frequency bands during an epileptic seizure (red shaded area indicates the clinically annotated ictal period). The analyzed window covers 100 s before seizure onset, the entire seizure, and 100 s after termination.

Figure 3 shows an increase in delta-band dWPLI during the ictal period. Delta oscillations are typically associated with slow large-scale cortical dynamics, reduced consciousness, and pathological synchronization [24]. Therefore, the increase in delta-band dWPLI suggests that slow oscillatory activity becomes more phase-stable and temporally coordinated across hemispheres during seizure propagation. An increase in dWPLI was also observed in the theta frequency band. Theta oscillations are frequently linked to long-range network coordination, limbic interactions, and transitional dynamical brain states [25,26]. Elevated theta-band dWPLI during seizures may therefore reflect enhanced recruitment and synchronization of distributed epileptic networks, potentially supporting seizure maintenance and propagation across large-scale brain systems.

### 2.4. Feature Extraction

For each EEG window, phase-based connectivity values were computed for all predefined channel pairs, frequency bands, and connectivity metrics described in Section 2.3. Connectivity values were analyzed separately for interhemispheric and intrahemispheric channel groups. Let $v_{i}^{\left(m,\Omega ,d\right)},\;i=1,\ldots ,6$, denote the connectivity values obtained for the six channel pairs within connectivity domain d∈{interhemispheric,intrahemispheric}, using connectivity metric m∈{ImagCoh,dwPLI} within frequency band Ω. To reduce dimensionality and obtain robust descriptors of synchronization structure, three summary statistics were computed across the six channel pairs for each combination of connectivity domain, frequency band, and connectivity metric.

The mean synchronization level was defined as

$$\mu^{\left(m,\Omega ,d\right)}=\frac{1}{6}\sum_{i=1}^{6}v_{i}^{\left(m,\Omega ,d\right)}.$$

This feature characterizes the overall synchronization strength within the corresponding connectivity domain.

The spatial variability of synchronization values across channel pairs was quantified using the standard deviation:

$$\sigma^{\left(m,\Omega ,d\right)}=\sqrt{\frac{1}{6}\sum_{i=1}^{6}{\left(v_{i}^{\left(m,\Omega ,d\right)}-\mu^{\left(m,\Omega ,d\right)}\right)}^{2}}.$$

This measure reflects the heterogeneity of synchronization patterns across spatially distributed channel pairs.

To capture extreme synchronization values, the maximum connectivity value across channel pairs was computed:

$$v_{\max}^{\left(m,\Omega ,d\right)}=\max_{i=1,\ldots ,6}v_{i}^{\left(m,\Omega ,d\right)}.$$

This feature emphasizes the strongest observed synchronization interaction within the analyzed connectivity domain.

Thus, for each frequency band, three features were obtained for each connectivity metric. Since two metrics (ImagCoh and dwPLI) were considered, this resulted in six features per frequency band. With four frequency bands (δ, θ, α, β), the total number of extracted features per EEG window was 4×6=24. These features characterize the distribution of phase-based synchronization values across channel pairs within each connectivity domain. These summary statistics capture complementary aspects of synchronization structure, including overall coupling strength (mean), spatial variability (standard deviation), and extreme synchronization values (maximum).

#### Feature Normalization

Prior to classification, the extracted feature vectors were standardized using z-score normalization. Let $v^{\left(m,\Omega \right)}$ denote a generic feature derived from the aggregation step (i.e., mean, standard deviation, or maximum defined above). The normalized feature is computed as

(6) $${v^{\left(m,\Omega \right)}}^{\prime }=\frac{v^{\left(m,\Omega \right)}-\mu_{train}^{\left(m,\Omega \right)}}{\sigma_{train}^{\left(m,\Omega \right)}},$$

where $\mu_{train}^{\left(m,\Omega \right)}$ and $\sigma_{train}^{\left(m,\Omega \right)}$ denote the mean and standard deviation of the corresponding feature estimated from the training data only.

The normalization is applied independently to each feature dimension. This ensures that all features have comparable scales, which improves the stability and convergence of neural network training. The same transformation parameters are subsequently applied to the test data to prevent information leakage.

### 2.5. Data Labeling

To evaluate temporal changes in synchronization during epileptic seizures, as well as at selected intervals before and after seizure onset, synchronization features were computed within a sliding time window of duration STW. The window was shifted relative to seizure onset according to predefined values of the parameter TAS (Time After Seizure onset):

TAS∈[−10,−5,0,5,10,15,20,30,40,50,60,70,80,90,100]s.

For each value of TAS, the analyzed interval was defined as

$$[t_{\mathrm{onset}}+TAS,\;t_{\mathrm{onset}}+TAS+STW].$$

Windows whose timestamps fell within this interval were assigned the label y(t)=1, whereas all remaining windows were labeled as baseline activity (y(t)=0). For each TAS value, a separate binary classification analysis was performed to distinguish seizure-related synchronization patterns from baseline synchronization activity. To reduce potential class imbalance caused by the substantially longer duration of baseline activity, balanced sampling was applied. Specifically, for each target interval, an equal number of baseline feature windows was randomly sampled from the baseline interval. For a given TAS, synchronization features were extracted from the interval $\left[t_{\mathrm{onset}}+TAS,\;t_{\mathrm{onset}}+TAS+STW\right]$. For example, TAS=−10 s with STW=30 s corresponds to the interval $\left[t_{\mathrm{onset}}-10,\;t_{\mathrm{onset}}+20\right]$ relative to seizure onset, including 10 s before and 20 s after seizure onset. Because the sliding window overlaps the seizure onset boundary, the analyzed interval may contain both preictal and early ictal activity. Similarly, for TAS=−500 s and STW=30 s, the corresponding target interval was $\left[t_{\mathrm{onset}}-500,\;t_{\mathrm{onset}}-470\right]$, representing activity occurring between 500 and 470 s before seizure onset. For each seizure recording, baseline activity was defined as the pre-onset interval

$$[t_{\mathrm{onset}}-600,\;t_{\mathrm{onset}}-30]\;s.$$

**Figure 4:**
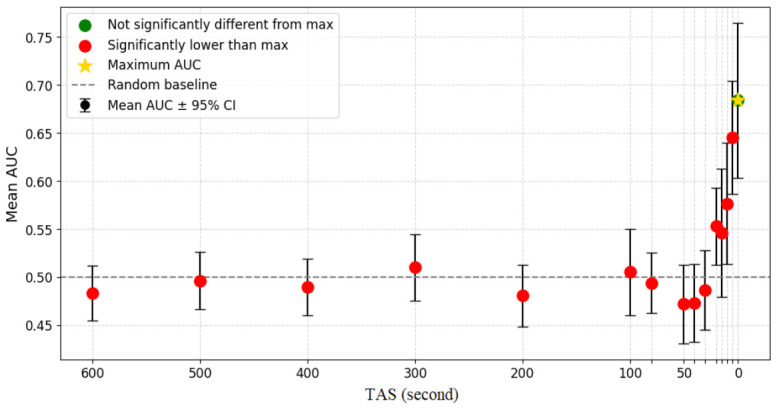
Dependence of the mean LOPO AUC on TAS for distant pre-onset baseline intervals.

Thus, for each target interval, an equal number of baseline feature windows was randomly sampled from this baseline interval. This interval was selected to exclude the immediate preictal transition period while preserving temporally proximal non-seizure activity from the same recording. Control analyses demonstrated that classification performance remained close to chance level for distant pre-onset intervals (e.g., TAS=−600 to −30 s), with mean AUC values approximately equal to 0.5. Figure 4 illustrates the dependence of the mean AUC obtained under the Leave-One-Patient-Out (LOPO) validation scheme as a function of TAS for the baseline analysis. The window was shifted relative to seizure onset according to the following predefined TAS values:

TAS∈[−600,−500,−400,−300,−200,−100,−80,−50,−40,−30,−20,−15,−10,−5,0]s.

For each value of TAS, the analyzed interval was defined as

$$[t_{\mathrm{onset}}+TAS,\;t_{\mathrm{onset}}+TAS+STW].$$

In contrast, classification separability progressively increased near seizure onset, beginning approximately 20–30 s before seizure onset and reaching maximal values during the ictal transition period. Importantly, the baseline interval was not interpreted as physiologically normal activity, since long-range preictal effects may extend substantially before seizure onset. Instead, the baseline represented temporally distant non-seizure activity used as a reference synchronization state for evaluating temporal deviations associated with seizure emergence.

This design enables the model to capture transitional synchronization dynamics occurring around seizure onset. The proposed labeling procedure allows the neural network model to learn how synchronization patterns within specific temporal intervals deviate from baseline activity. To determine the optimal window length, values of STW ranging from 5 to 50 s were systematically evaluated. Model performance was assessed using the Leave-One-Patient-Out (LOPO) cross-validation scheme. The highest AUC values were obtained for STW=30 s, indicating that this window length provides the best discrimination between seizure-related and baseline synchronization patterns. A more detailed analysis of the influence of STW is provided in Appendix A. Therefore, STW=30 s was selected for the final analysis.

### 2.6. Neural Network Architecture

Classification was performed using a multilayer perceptron (MLP). Let $x\in R^{24}$ denote the input feature vector constructed from the aggregated synchronization features derived from Imaginary Coherence and dwPLI across all frequency bands (see Section 2.5). Let y∈{0,1} denote the corresponding class label, where y=1 indicates that the EEG window belongs to a seizure-related interval, and y=0 corresponds to baseline (interictal) activity. Importantly, the connectivity measures (ImagCoh and dwPLI) are used as input features, whereas *y* represents the target variable to be predicted by the classifier. The network consists of a fully connected hidden layer with 64 neurons followed by an output layer with two units representing class probabilities. The hidden layer is defined as

(7) $$h=\mathrm{ReLU}(W_{1}x+b_{1}),$$

where $W_{1}\in R^{64\times 24}$ and $b_{1}\in R^{64}$ denote the weight matrix and bias vector. The output layer produces class probabilities using the softmax function:

(8) $$\hat{y}=\mathrm{softmax}\left(W_{2}h+b_{2}\right),$$

where $\hat{y}=\left({\hat{y}}_{0},{\hat{y}}_{1}\right)$ represents the estimated probabilities of the two classes. In particular, ${\hat{y}}_{1}=P\left(y=1|x\right)$ denotes the predicted probability that a given EEG window corresponds to seizure-related activity.

This relatively shallow architecture was selected to balance model expressiveness and robustness given the limited dataset size. Deep neural networks with many layers are susceptible to the vanishing gradient problem, whereby gradients diminish exponentially during backpropagation and impede convergence of early layers [27,28]. In the present work, this issue is mitigated by design: the shallow MLP architecture (a single hidden layer of 64 neurons with ReLU activations) avoids the depth at which vanishing gradients typically become severe. ReLU activations preserve gradient magnitude for positive pre-activations, and the combination of Adam optimisation with L2 regularisation further stabilises training without requiring specialised gradient-correction strategies.

#### 2.6.1. Training Procedure

Model parameters were optimized using the Adam algorithm, an adaptive stochastic gradient-based optimization method. The network was trained to minimize the binary cross-entropy loss:

(9) $$L=-\left[y\log\left({\hat{y}}_{1}\right)+\left(1-y\right)\log\left({\hat{y}}_{0}\right)\right].$$

To reduce overfitting, L2 regularization was applied to the network weights:

(10) $$L_{\mathrm{reg}}=L+\alpha {∥W∥}_{2}^{2},$$

where ${∥W∥}_{2}^{2}$ denotes the sum of squared weights across all layers, and α is a regularization coefficient. The value of α was selected based on validation performance. Training was performed for a maximum of 500 epochs with early stopping. Training was terminated if validation performance did not improve for 10 consecutive epochs. This strategy mitigates overfitting and improves generalization.

#### 2.6.2. Performance Evaluation

Model performance was evaluated using the Area Under the Receiver Operating Characteristic Curve (AUC-ROC). For each EEG window, the trained model outputs the predicted probability ${\hat{y}}_{1}=P\left(y=1|x\right)$, where y∈{0,1} denotes the true class label. These probabilities were used to construct the ROC curve by plotting the True Positive Rate (TPR) against the False Positive Rate (FPR) across varying decision thresholds. The AUC quantifies the ability of the model to discriminate between seizure-related (y=1) and baseline (y=0) EEG windows. AUC values range from 0.5 (random classification) to 1.0 (perfect discrimination). Within the Leave-One-Patient-Out (LOPO) framework, the AUC was computed separately for each held-out patient:

(11) $${\mathrm{AUC}}_{k}=\mathrm{AUC}\left({\hat{y}}_{1}^{\left(k\right)},y^{\left(k\right)}\right),$$

where ${\hat{y}}_{1}^{\left(k\right)}$ denotes the predicted probabilities and $y^{\left(k\right)}$ the true labels for patient *k*. The overall performance was then summarized by averaging across patients:

(12) $${\mathrm{AUC}}_{\mathrm{mean}}=\frac{1}{N}\sum_{k=1}^{N}{\mathrm{AUC}}_{k},$$

where *N* is the total number of patients.

### 2.7. Validation Strategy: Leave-One-Patient-Out

To evaluate inter-patient generalization performance, a Leave-One-Patient-Out (LOPO) cross-validation scheme was adopted.

Assuming *N* patients in the dataset, the procedure consists of *N* iterations. At each iteration:

- All recordings from one patient are used exclusively for testing.
- Recordings from the remaining N−1 patients are used for training.

This approach prevents information leakage between patients and provides a clinically realistic estimate of model generalizability to unseen subjects. Performance metrics were computed separately for each patient and subsequently averaged across patients.

### 2.8. Definition of Relative Pathological Synchronization

Within the proposed framework, synchronization dynamics were quantified using a model-based measure derived from classification performance. For each temporal condition defined by the parameter TAS, a separate classifier was trained to distinguish synchronization features extracted from the corresponding target interval from baseline activity. Classification performance was evaluated using the Leave-One-Patient-Out (LOPO) validation strategy described above. Let $AUC_{k}\left(TAS\right)$ denote the classification performance obtained for the held-out patient *k* at a given temporal offset TAS.

The Relative Pathological Synchronization (RPS) was defined as the average LOPO classification performance across all patients:

(13) $$RPS\left(TAS\right)=\frac{1}{N}\sum_{k=1}^{N}AUC_{k}\left(TAS\right),$$

where *N* denotes the total number of patients.

Within this formulation, larger values of RPS(TAS) indicate that synchronization features extracted from the corresponding temporal interval are more consistently distinguishable from baseline activity across subjects. Importantly, RPS should not be interpreted as a direct physiological or biophysical measure of neuronal synchronization strength. Instead, it represents a model-dependent measure of synchronization-related separability under a fixed feature representation, classification model, and validation protocol. More specifically, RPS quantifies the extent to which large-scale synchronization patterns support reliable patient-independent discrimination between seizure-related and baseline EEG activity. Under this interpretation, the resulting RPS(TAS) curve provides a compact representation of the temporal evolution of pathological synchronization-related network organization relative to seizure onset. Because the RPS(TAS) curve is derived from classification performance, its numerical values depend on the selected connectivity measures, feature extraction procedure, classifier architecture, and validation framework. Consequently, RPS should be interpreted as a relative and framework-specific indicator rather than an intrinsic physiological quantity.

## 3. Results

This section presents the experimental results obtained from the proposed RPS framework applied to the CHB-MIT database. Section 3.1 examines the temporal evolution of RPS for interhemispheric and intrahemispheric connectivity. Section 3.2 investigates the relationship between seizure duration and peak RPS values. Section 3.3 analyses permutation-based feature importance to identify the most informative frequency bands and connectivity measures. Section 3.4 provides a statistical ablation comparison across individual frequency bands.

All experiments and model training were conducted in Google Colab (cloud environment) using Python 3.10. The computations utilized an NVIDIA Tesla T4 GPU with 16 GB VRAM, an Intel Xeon CPU, and 52 GB of system RAM. The main libraries used include NumPy, SciPy, scikit-learn, MNE-Python, PyTorch 2.1, and Matplotlib (version 3.10.0) for visualization. All models were trained using the Adam optimizer with early stopping, and results were obtained under strict Leave-One-Patient-Out cross-validation.

### 3.1. Temporal Evolution of RPS for Interhemispheric and Intrahemispheric Connectivity

**Figure 5:**
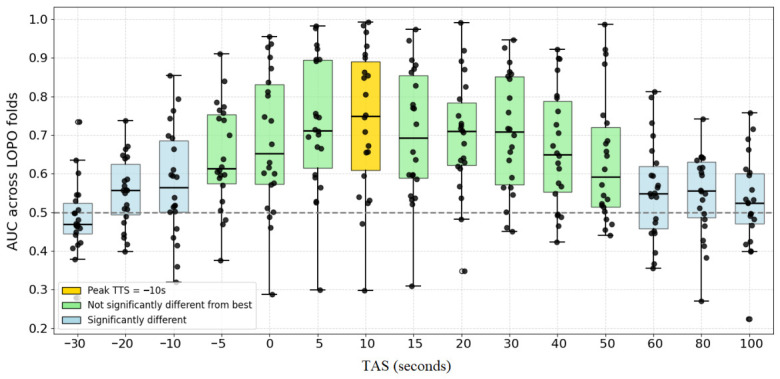
Distribution of patient-specific interhemispheric RPS across LOPO folds for different TAS values.

We analyzed the temporal evolution of Relative Pathological Synchronization RPS(TAS) for interhemispheric and intrahemispheric connectivity features, where RPS is defined as the mean AUC obtained under the Leave-One-Patient-Out (LOPO) validation scheme. Figure 5 illustrates the distribution of patient-specific AUC values across LOPO folds for different TAS values. Boxplots show the median, interquartile range, and outliers, while individual points correspond to held-out patient results.

The results demonstrate that for interhemispheric connectivity, RPS(TAS) increases sharply before seizure onset, reaching a maximum at approximately TAS=10 s, followed by a plateau in the interval TAS∈[−5,50] s (Wilcoxon signed-rank test, p>0.05), where −5 s denotes 5 s before attack. Within this interval, synchronization patterns are most consistently distinguishable from baseline activity across patients, indicating a highly reproducible deviation of network organization from interictal dynamics.

From an algorithmic perspective, this behaviour reflects the sensitivity of the MLP classifier to the phase-lag-based connectivity features extracted during the early ictal period. The sharp rise in AUC near TAS=0 indicates that the 24-dimensional feature vector—derived from ImagCoh and dwPLI across four frequency bands—undergoes a rapid and consistent redistribution relative to the baseline distribution as the seizure begins. The plateau at TAS∈[−5,50] s suggests that the decision boundary learned by the classifier remains stable throughout the early-to-mid ictal phase, implying that the network enters a sustained pathological synchronization regime that is reproducible across patients. The widening of the interquartile range at later time points (TAS>40 s) reflects increasing inter-patient variability, consistent with the heterogeneous termination dynamics of epileptic seizures. The gradual return of the median AUC toward chance level (≈0.5) beyond TAS≈60 s indicates that the classifier, trained on population-level statistics, loses discriminative power as post-ictal connectivity patterns converge back to baseline.

**Figure 6:**
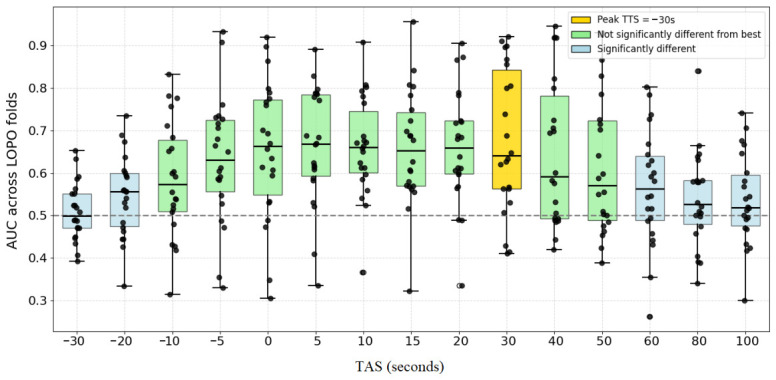
Distribution of patient-specific intrahemispheric RPS across LOPO folds for different TAS values.

Figure 6 presents the corresponding AUC values across LOPO folds for different TAS values, obtained from the classifier trained on intrahemispheric connectivity features.

In contrast to interhemispheric connectivity, intrahemispheric connectivity exhibits a delayed maximum of RPS(TAS) at approximately TAS=30 s and a broader plateau extending from TAS=−10 s to TAS=50 s. This suggests that deviations of intrahemispheric synchronization patterns from baseline evolve more gradually and remain detectable over a longer temporal interval. A direct comparison shows that interhemispheric connectivity achieves higher peak RPS values than intrahemispheric connectivity ($Q_{2}$ ($Q_{1}$–$Q_{3}$): 0.749 (0.609–0.891) vs. 0.640 (0.563–0.843), p<0.05), indicating that interhemispheric synchronization patterns provide more consistent and robust discrimination from baseline activity across patients.

From an algorithmic standpoint, the delayed and broader peak observed for intrahemispheric features reflects a fundamentally different temporal structure of the learned decision boundary. The MLP classifier trained on intrahemispheric connectivity features requires a longer accumulation of ictal activity before the feature distribution separates reliably from baseline. This is consistent with the nature of intrahemispheric coupling: ipsilateral channel pairs sample spatially proximate regions within the same hemisphere, and pathological synchronization in these pathways may develop more gradually as the seizure recruits local cortical networks. The broader plateau (TAS∈[−10,50] s) implies that the classifier maintains a stable decision boundary over an extended ictal window, suggesting that intrahemispheric synchronization is sustained throughout much of the seizure duration rather than peaking sharply at onset. The larger interquartile range compared to the interhemispheric model further reflects that local synchronization patterns are more patient-specific, making population-level generalization inherently harder. Beyond TAS≈40–60 s, RPS(TAS) decreases toward chance level (≈0.5) for both connectivity domains, reflecting a progressive reduction in the distinguishability of synchronization patterns relative to interictal activity.

### 3.2. Relationship Between Seizure Duration and RPS

**Figure 7:**
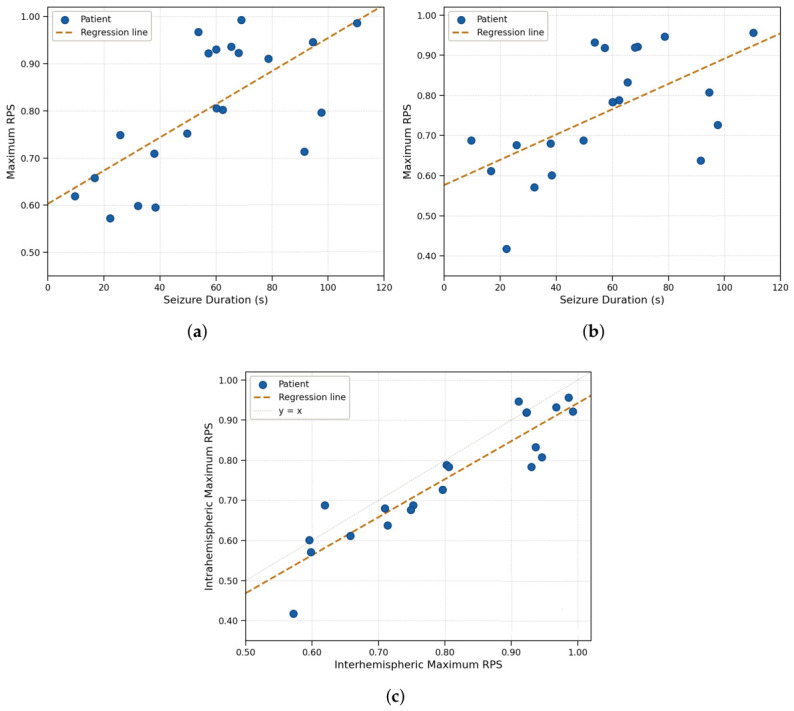
(**a**) Scatter plot of seizure duration versus peak interhemispheric RPS across patients. The dashed line shows the linear regression fit. Spearman’s ρ=0.684, p=0.0006. (**b**) Scatter plot of seizure duration versus peak intrahemispheric RPS across patients. The dashed line shows the linear regression fit. Spearman’s ρ=0.651, p=0.0014. (**c**) Scatter plot of peak interhemispheric RPS versus peak intrahemispheric RPS across patients. The dashed line shows the linear regression fit; the dotted line indicates the identity (y=x), highlighting that interhemispheric RPS values are systematically higher. Spearman’s ρ=0.911, p<0.0001.

We examined the relationship between seizure duration and the maximum RPS value achieved across patients. Figure 7 illustrates the corresponding scatter plots with regression lines.

A significant positive association was observed for both the interhemispheric model (Spearman’s ρ=0.684, p=0.0006) and the intrahemispheric model (Spearman’s ρ=0.651, p=0.0014), indicating that longer seizures tend to be associated with higher peak RPS values (Figure 7a,b). This effect cannot be attributed to differences in window length, as the sliding time window STW was fixed at 30 s across all seizures. Instead, the results suggest that longer seizures produce synchronisation patterns that deviate more consistently from baseline activity across patients, leading to improved population-level separability.

A strong positive correlation was also observed between the interhemispheric and intrahemispheric peak RPS values (ρ=0.911, p<0.0001; Figure 7c), indicating that patients with highly separable interhemispheric synchronisation patterns also tend to exhibit strongly separable intrahemispheric patterns. This co-variation suggests that both connectivity domains reflect a common underlying large-scale network deviation from baseline dynamics, rather than independent localised processes.

### 3.3. Feature Importance and Frequency-Specific Contributions

**Figure 8:**
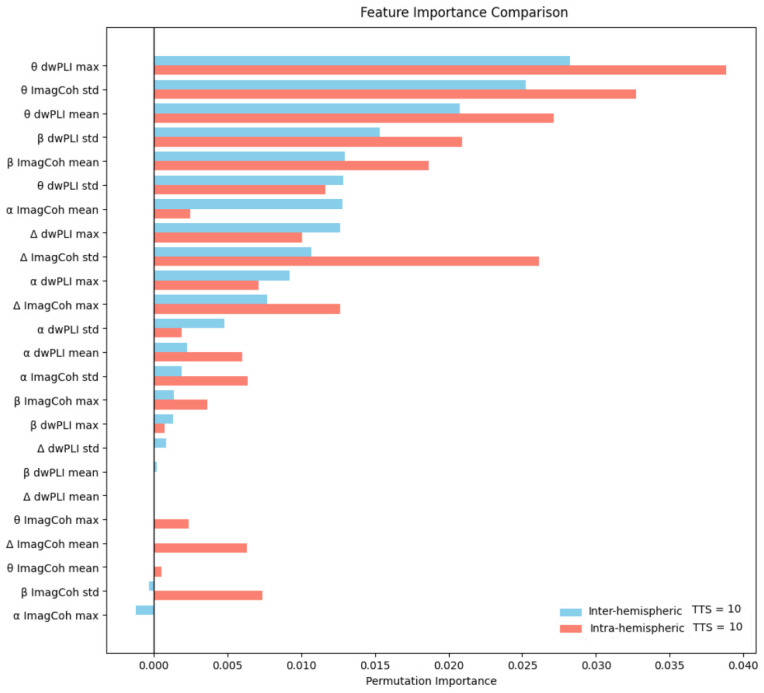
Feature importance comparison based on permutation importance at TAS=10 s.

Permutation feature importance analysis was employed to rank the most informative features contributing to model performance. The analysis was conducted at time points corresponding to maximal synchronization changes, as determined by the peak RPS values for each model. For the interhemispheric model, the maximum RPS was observed at TAS=10 s. Accordingly, permutation feature importance was evaluated at this time point using the corresponding AUC values. Figure 8 illustrates the ranking of the most informative features for the interhemispheric model (blue), alongside the intrahemispheric model (red) for comparison at TAS=10 s.

**Figure 9:**
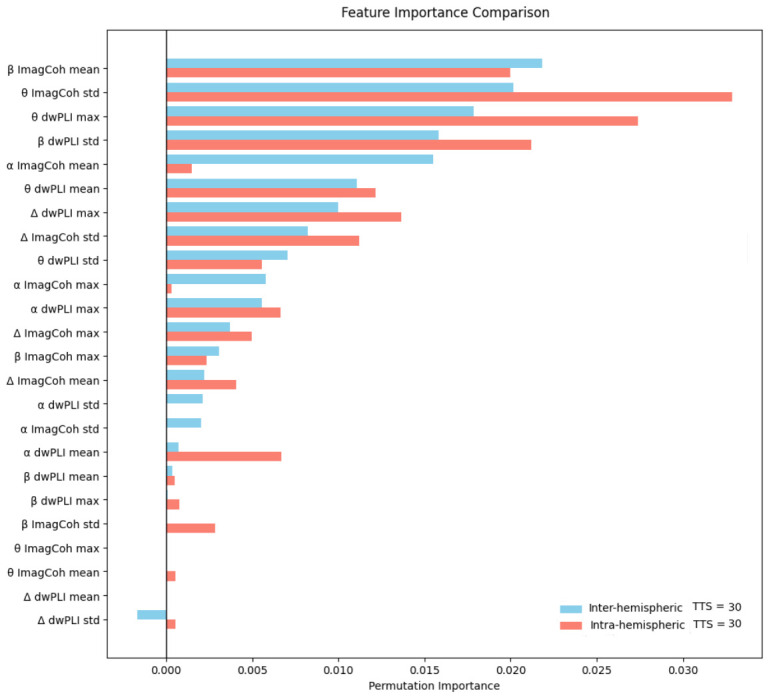
Feature importance comparison based on permutation importance at TAS=30 s.

As shown in Figure 8, the most informative features for the interhemispheric model at peak RPS are dominated by theta-band connectivity measures, specifically $\theta {\mathrm{dwPLI}}_{\max}$, $\theta {\mathrm{ImagCoh}}_{std}$, and $\theta {\mathrm{dwPLI}}_{mean}$. This indicates a prominent role of phase-based synchronization metrics in the theta band for interhemispheric interactions. For the intrahemispheric model, the maximum RPS was observed at TAS=30 s. The corresponding feature importance rankings are presented in Figure 9, which compares intrahemispheric (red) and interhemispheric (blue) models at TAS=30 s.

From an algorithmic perspective, the dominance of $\theta {\mathrm{dwPLI}}_{\max}$ reflects the sensitivity of the classifier to the most extreme synchronization value observed across the six interhemispheric channel pairs. Because the MLP uses z-score-normalized features, permuting this feature disrupts the largest variance direction in the input space, producing the greatest degradation in AUC. The prominence of $\theta {\mathrm{ImagCoh}}_{std}$ indicates that the temporal variability of phase-lagged coherence—rather than its mean level—is a primary discriminant between seizure-related and baseline windows. Algorithmically, this implies that the classifier has learned to separate distributions that differ principally in their spread rather than their central tendency, which is consistent with the non-stationary and reconfiguring nature of ictal connectivity dynamics.

At this time point, the intrahemispheric model is primarily driven by $\theta {\mathrm{ImagCoh}}_{std}$ and $\theta {\mathrm{dwPLI}}_{\max}$, with an additional contribution from $\beta {\mathrm{dwPLI}}_{std}$. This suggests that while theta-band synchronization remains dominant, higher-frequency beta-band variability may provide complementary information for intrahemispheric connectivity. The feature importance results indicate a clear dominance of theta-band phase-synchronization measures, specifically $\theta {\mathrm{dwPLI}}_{\max}$, $\theta {\mathrm{ImagCoh}}_{std}$, and $\theta {\mathrm{dwPLI}}_{mean}$ for the interhemispheric model, and $\theta {\mathrm{ImagCoh}}_{std}$ together with $\theta {\mathrm{dwPLI}}_{\max}$ for the intrahemispheric model, with an additional contribution from $\beta {\mathrm{dwPLI}}_{std}$.

The prominence of $\theta {\mathrm{dwPLI}}_{\max}$ suggests that transient changes in synchronization are particularly informative. In other words, the model is sensitive to time periods when connectivity deviates from its baseline level, rather than relying only on average connectivity. This is consistent with the established role of theta oscillations in coordinating large-scale brain activity across distributed regions [29]. At the same time, the contribution of $\theta {\mathrm{dwPLI}}_{mean}$ (in the interhemispheric model) indicates that the overall level of synchronization also plays a role. Together, these results suggest that interhemispheric communication is characterized by both baseline connectivity and its transient deviations during seizure-related dynamics.

The consistent importance of $\theta {\mathrm{ImagCoh}}_{std}$ highlights that variability of connectivity over time is a key factor. This implies that not only the magnitude of synchronization, but also its temporal fluctuations, carry relevant information. Such variability likely reflects dynamic reconfiguration of functional networks. Importantly, imaginary coherence captures phase-lagged interactions and reduces the influence of volume conduction, making it a more reliable indicator of true inter-regional communication [10]. For the intrahemispheric model, the additional contribution of $\beta {\mathrm{dwPLI}}_{std}$ suggests that higher-frequency dynamics provide complementary information, potentially reflecting local processing within cortical regions [21,22].

Overall, the results support an interpretation in which theta-band synchronization reflects large-scale coordination between brain regions, while variability in both theta and beta bands captures the dynamic, time-varying nature of functional connectivity during seizure-related states.

Algorithmically, the reduced set of important features at TAS=30 s for the intrahemispheric model indicates that the classifier relies on a sparser representation of the input space at this time point. This sparsity may reflect a more localised and frequency-specific reorganisation of intrahemispheric networks during mid-ictal activity, in which theta-band dynamics alone provide sufficient separability. The additional contribution of $\beta {\mathrm{dwPLI}}_{std}$ suggests that the variability of fast intrahemispheric synchronization provides complementary information not captured by theta-band features alone, potentially corresponding to local cortical oscillatory dynamics associated with sustained seizure activity.

### 3.4. Statistical Comparison Across Frequency Bands

To quantify the relative contribution of individual frequency bands, we performed pairwise statistical comparisons between all ablation configurations using paired *t*-tests. The analysis was conducted at time points corresponding to maximal changes in synchronization, as identified by the peak RPS values for each model. For the interhemispheric model, the maximum RPS was observed at TAS=10 s. Therefore, the ablation analysis was performed on the corresponding AUC values to assess the contribution of each frequency band during the most pronounced synchronization changes associated with seizure dynamics. Table 1 shows the mean AUC values and corresponding 95% confidence intervals for the full model and when one of the frequency bands is removed.

All ablation configurations resulted in a decrease in performance relative to the full model. The largest reduction was observed after removal of the alpha band (mean AUC: 0.680 vs. 0.738 for the full model), followed by the beta (0.699) and theta (0.692) bands (Table 1).

From an algorithmic perspective, these results reflect the contribution of each frequency band to the separability of the 24-dimensional feature distribution. The full model benefits from a richer feature space in which connectivity patterns across multiple bands provide complementary discriminative information. Removing any single band reduces the effective dimensionality of the input and eliminates features that capture distinct aspects of ictal synchronization dynamics. The fact that alpha removal produces the largest mean performance drop suggests that alpha-band phase-lag connectivity undergoes particularly consistent changes across patients at TAS=10 s. This may reflect the well-documented suppression or reorganization of alpha-band activity during seizure onset, which generates a consistent shift in the feature distribution that the MLP can reliably exploit.

Table 2 shows the pairwise comparison of ablation interhemispheric models.

Pairwise statistical comparisons confirmed that removal of each frequency band led to a statistically significant degradation in performance relative to the full model (p<0.05 in all cases; Table 2). In contrast, comparisons between ablation conditions did not reveal statistically significant differences (p>0.05), indicating that no single band can be considered strictly dominant in isolation.

Algorithmically, the lack of significant differences between ablation conditions implies that the classifier distributes its discriminative reliance across multiple frequency bands rather than depending on any single one. This is consistent with the architecture of the MLP, which learns a joint linear combination of all input features in the hidden layer; removing one band perturbs the learned weight matrix but does not collapse performance because the remaining bands provide partially redundant information. The overall pattern supports a distributed, multi-band mechanism underlying interhemispheric seizure-related synchronization, in which the combined contribution of theta, alpha, and beta bands exceeds that of any individual band.

For the intrahemispheric model, the maximum RPS was observed at TAS=30 s, and the corresponding AUC values were used for the ablation analysis. Table 3 summarizes the mean AUC values and corresponding 95% confidence intervals for the full model and each ablation condition.

As shown in Table 3, all ablation conditions produced a decrease in mean AUC relative to the full model (0.696). The largest reduction was associated with removal of the theta band (mean AUC: 0.661), while alpha (0.671) and beta (0.675) removal produced smaller decrements.

From an algorithmic standpoint, the larger impact of theta removal on intrahemispheric classification compared to alpha or beta removal suggests that theta-band features occupy a more central role in the learned decision boundary at TAS=30 s. Because the MLP hidden layer computes a nonlinear transformation of the full 24-dimensional input, features with higher variance or greater class-conditional separation receive implicitly larger effective weights. Theta-band connectivity measures—particularly $\theta {\mathrm{ImagCoh}}_{std}$ and $\theta {\mathrm{dwPLI}}_{\max}$—likely exhibit larger distributional shifts between seizure-related and baseline windows for intrahemispheric pairs at this time point, making them primary drivers of the classification boundary.

This observation is further supported by the pairwise comparisons presented in Table 4.

A significant difference was found only for the comparison between the full model and the no_theta condition (p=0.038), while all other comparisons were not statistically significant (p>0.05; Table 4). These results indicate a more prominent role of theta-band activity in intrahemispheric connectivity patterns during seizure evolution.

Algorithmically, the non-significant effect of alpha and beta removal implies that these bands provide features whose class-conditional distributions do not differ substantially from baseline at the population level for intrahemispheric pairs at TAS=30 s. This may reflect the fact that higher-frequency oscillatory dynamics in ipsilateral cortical pathways are more patient-specific and therefore do not contribute consistently to a shared population-level decision boundary. In contrast, theta-band dynamics appear to reflect a more universal mechanism of intrahemispheric ictal synchronization, one that is reproducible enough across patients to constitute a statistically reliable discriminant feature under LOPO validation. The observed frequency-specific effects are broadly consistent with prior literature on EEG-based seizure analysis [1,11,30].

## 4. Discussion

Unlike detection-oriented approaches, the present study addresses a complementary objective: rather than optimising segment-level classification performance, we aim to characterise *how* and *when* large-scale synchronisation patterns deviate from baseline activity in a reproducible, patient-independent manner. To this end, we introduced Relative Pathological Synchronisation (RPS) as a model-derived indicator of population-level separability, evaluated under a strict LOPO validation framework. Although RPS is not directly optimised for detection, it is informative to place the obtained AUC values in the context of contemporary patient-independent methods on the CHB-MIT dataset. Table 5 summarises this comparison, distinguishing between detection-oriented and characterisation-oriented objectives.

Table 5 places the proposed RPS framework in the context of contemporary patient-independent methods evaluated on the CHB-MIT dataset. Only studies employing a strict Leave-One-Patient-Out (LOPO) or Leave-One-Subject-Out (LOSO) validation scheme are included, as these represent the most directly comparable evaluation protocols.

As shown in Table 5, existing patient-independent methods achieve high detection performance, with sensitivity up to 0.95 and AUC ≥ 0.99 under LOSO validation [31]. The RPS framework reports lower AUC values (0.75 (0.61–0.89) and 0.64 (0.56–0.84) for interhemispheric and intrahemispheric connectivity, respectively), but this difference reflects the fundamentally different objective: RPS quantifies the consistency of synchronisation-related network changes across patients at specific time points relative to seizure onset, rather than optimising segment-level classification performance. Importantly, unlike detection-oriented methods, the RPS framework additionally reveals *when* pathological connectivity patterns emerge (approximately 5 s before clinical onset for interhemispheric connectivity), which frequency bands contribute most (theta-band dominance), and how these dynamics relate to seizure duration.

A key finding of the present study is the temporal dissociation between interhemispheric and intrahemispheric synchronization. Interhemispheric connectivity exhibited earlier and more pronounced deviations from baseline, whereas intrahemispheric dynamics evolved more gradually and reached peak separability at later time points. This pattern suggests that seizure evolution involves an early engagement of large-scale bilateral interactions followed by more sustained intrahemispheric reorganization. Such observations are consistent with the conceptualization of epilepsy as a network disorder involving distributed and dynamically evolving interactions across brain regions [3,4].

The analysis of frequency-specific contributions reveals a more nuanced picture. Feature importance consistently identified theta-band synchronization measures (particularly $\theta {\mathrm{dwPLI}}_{\max}$ and $\theta {\mathrm{ImagCoh}}_{std}$) as highly informative across both connectivity domains. These features capture transient deviations from baseline connectivity as well as temporal variability, indicating that seizure-related dynamics are characterized by non-stationary and reconfiguring network interactions.

However, the statistical ablation analysis indicates that the contribution of individual frequency bands is not uniform across connectivity domains. For interhemispheric connectivity, removal of any frequency band (theta, alpha, or beta) led to a significant decrease in performance, while no single band was statistically dominant over the others. This suggests that interhemispheric synchronization during seizures is supported by a distributed, multi-band mechanism. Such broadband involvement is consistent with previous studies reporting that epileptic activity engages multiple frequency ranges and may involve cross-frequency interactions [1,11].

In contrast, intrahemispheric connectivity exhibited a more specific dependence on the theta band, with only theta removal leading to a statistically significant degradation in performance. This indicates that theta-band dynamics may play a more prominent role in local or within-hemisphere synchronization processes during seizure evolution. Theta oscillations have been widely associated with large-scale coordination and long-range communication [26,32], but their role in structuring local network interactions has also been reported, particularly in the context of pathological synchronization [2].

Taken together, these findings suggest a hierarchical and multi-scale organization of seizure-related connectivity. Interhemispheric interactions appear to rely on a broadband synchronization structure, while intrahemispheric dynamics show a relatively stronger dependence on theta-band processes. Importantly, the prominence of variability-based features (e.g., $\theta {\mathrm{ImagCoh}}_{std}$) further indicates that the temporal fluctuations of connectivity, rather than static synchronization levels, are critical for capturing seizure-related network changes. This observation aligns with prior work emphasizing the dynamic and non-stationary nature of epileptic brain networks [1,2].

Another important result is the positive association between seizure duration and peak RPS values. Longer seizures were associated with higher classification performance, suggesting that synchronization patterns become more consistently distinguishable from baseline activity as the seizure progresses. This may reflect progressive stabilization of pathological network configurations during ictal evolution, a phenomenon that has been observed in both experimental and clinical studies [7,33].

From a methodological perspective, the use of phase-lag-based connectivity measures (ImagCoh and dwPLI) reduces the influence of volume conduction and emphasizes delayed interactions between neural sources [10,23]. In addition, the proposed RPS framework provides a model-based characterization of synchronization dynamics that captures the collective structure of connectivity features under a patient-independent validation scheme. This is particularly important given the substantial inter-patient variability reported in epilepsy studies [30].

Several limitations should be acknowledged. First, the use of a reduced set of EEG channels may limit spatial resolution. Second, RPS is a model-dependent measure and does not directly quantify physiological synchronization, but rather the separability of connectivity patterns. Third, cross-frequency interactions were not explicitly modeled and may contribute additional information.

Future work may extend this approach by incorporating higher-density EEG, exploring additional frequency bands and cross-frequency interactions [12], cross-scale interactions in wavelets analysis [34] and information-theoretic cross-oscillatory interactions [35], and applying similar methodology to seizure prediction tasks. Overall, the present findings support a network-based view of epilepsy and highlight the importance of both multi-band and frequency-specific mechanisms in shaping seizure-related functional connectivity.

## 5. Conclusions

In this study, we analyzed large-scale EEG synchronization during epileptic seizures using a patient-independent framework based on phase-lag-based connectivity measures. A key strength of the proposed approach is the use of a strict Leave-One-Patient-Out (LOPO) validation scheme, which ensures that the reported results reflect true inter-patient generalizability rather than subject-specific patterns. We introduced the Relative Pathological Synchronization (RPS) as a model-based indicator that captures the population-level separability between seizure-related and baseline network dynamics. Within this framework, we identified robust and reproducible synchronization signatures that are consistent across patients, supporting the network-based view of epilepsy. Overall, the results demonstrate that large-scale synchronization patterns contain stable, patient-independent information about seizure dynamics. These findings highlight the potential of connectivity-based representations, combined with rigorous validation strategies, for developing clinically relevant and generalizable EEG analysis methods.

Quantitatively, interhemispheric connectivity achieved a peak RPS=0.749 (0.609–0.891) at TAS=10 s, while intrahemispheric connectivity reached RPS=0.640 (0.563–0.843) at TAS=30 s, both under strict LOPO validation. A significant positive correlation between seizure duration and peak RPS was observed for both connectivity domains (Spearman’s ρ=0.684 and 0.651, respectively, p<0.005), and theta-band features were identified as the most consistent contributors across patients.

## Figures and Tables

**Table 1:** Mean AUC with 95% confidence intervals for the interhemispheric model.

Method	Mean AUC	95% CI
all	0.738	[0.656, 0.820]
no_theta	0.692	[0.612, 0.772]
no_alpha	0.680	[0.604, 0.756]
no_beta	0.699	[0.625, 0.773]

**Table 2:** Pairwise comparison of ablation interhemispheric models.

Comparison	*p*-Value
all vs. no_theta	0.021
all vs. no_alpha	0.001
all vs. no_beta	0.029
no_theta vs. no_alpha	0.42
no_theta vs. no_beta	0.63
no_alpha vs. no_beta	0.16

**Table 3:** Mean AUC with 95% confidence intervals for the intrahemispheric model.

Method	Mean AUC	95% CI
all	0.696	[0.635, 0.757]
no_theta	0.661	[0.595, 0.727]
no_alpha	0.671	[0.609, 0.733]
no_beta	0.675	[0.608, 0.742]

**Table 4:** Pairwise comparison of ablation intrahemispheric models.

Comparison	*p*-Value
all vs. no_theta	0.038
all vs. no_alpha	0.11
all vs. no_beta	0.23
no_theta vs. no_alpha	0.46
no_theta vs. no_beta	0.37
no_alpha vs. no_beta	0.78

**Table 5:** Patient-independent methods on CHB-MIT (LOPO/LOSO only). D = Detection; C = Characterisation Reported metrics follow original publications (AUC, accuracy, or F1-score). “–” indicates that the metric was not reported.

Method (Ref.)	Year	Sens.	Spec.	AUC / F1 / Acc.	Obj.
CNN + BiLSTM [15]	2022	0.86	–	0.91 (AUC)	D
Ensemble GBM [31]	2026	0.95	–	0.99 (AUC)	D
CA-EEGWaveNet [18]	2026	–	–	0.78 (F1)	D
RPS inter-hemi. (ours)	2026	0.67	0.64	0.75 (0.61–0.89) (AUC)	C
RPS intra-hemi. (ours)	2026	0.39	0.80	0.64 (0.56–0.84) (AUC)	C

**Figure A1:**
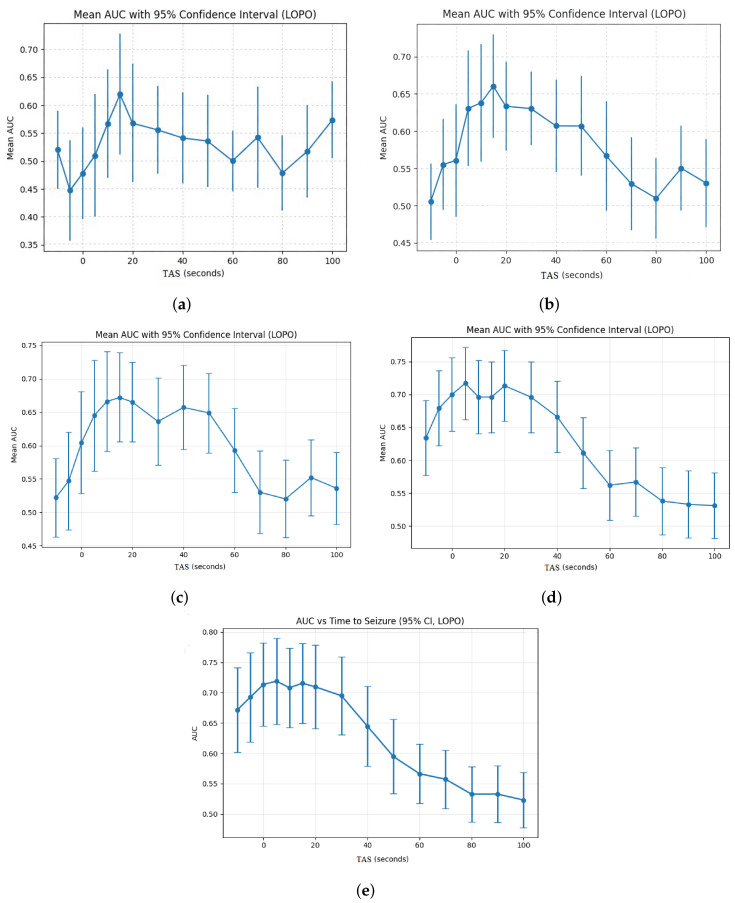
Mean AUC with 95% Confidence Interval (LOPO) for (**a**) STW = 5 s. (**b**) STW = 15 s. (**c**) STW = 20 s. (**d**) STW = 40 s. (**e**) STW = 50 s.

## Data Availability

The original data presented in the study are openly available in PhysioNet at https://physionet.org/content/chbmit/1.0.0/ (accessed on 14 April 2026).

## Appendix A. Additional STW Analysis

To determine the optimal value of the sliding time window (STW), we evaluated the area under the ROC curve (AUC) for several window lengths: 5, 15, 20, 30, 40, and 50 s. The AUC values were estimated using the Leave-One-Patient-Out (LOPO) cross-validation scheme.

Analyzing Figure 5 and Figure A1, we see that the maximum AUC is achieved for STW = 30 at TAS = 10.
